# Evaluating Tumor Regression and Survival Outcomes in Pancreatic Ductal Adenocarcinoma After Neoadjuvant Treatment according to CAP Grading System: Clinical Usefulness and Limitations

**DOI:** 10.1245/s10434-025-18123-w

**Published:** 2025-08-26

**Authors:** Go-Won Choi, Younsoo Seo, Inhyuck Lee, Yoon Soo Chae, Won-Gun Yun, Youngmin Han, Hye-Sol Jung, Young Jae Cho, Wooil Kwon, Joon Seong Park, Jin-Young Jang

**Affiliations:** Department of Surgery and Cancer Research Institute, Seoul National University College of Medicine, Seoul, Republic of Korea

**Keywords:** Pancreatic cancer, Neoadjuvant treatment, Tumor regression grading, CAP grade, Pathologic complete response

## Abstract

**Background:**

Neoadjuvant treatment (NAT) is increasingly employed for pancreatic ductal adenocarcinoma (PDAC), necessitating reliable methods to assess tumor response. The College of American Pathologists (CAP) grading system is widely used to predict chemo-responsiveness and survival, but consensus on the most effective grading system and treatment regimen remains elusive.

**Patients and Methods:**

This retrospective study included 462 patients with PDAC who underwent resection after NAT between 2009 and 2023. Survival outcomes were analyzed on the basis of CAP grades, and factors influencing favorable tumor responses were identified.

**Results:**

Patients with CAP grades 0 and 1 showed significantly better overall survival (OS) and disease-free survival (DFS) than those with CAP grades 2 and 3. Key factors associated with improved outcomes included the 5-fluorouracil, irrinotecan, leucovorin, and oxaliplatin (FOLFIRINOX) regimen, stereotactic ablative body radiotherapy (SABR), and adjuvant chemotherapy. Despite achieving a pathological complete response, younger patients and those with pre-NAT suspected metastases were more prone to recurrence.

**Conclusions:**

CAP grade is a strong prognostic tool for PDAC after NAT. Survival outcomes are enhanced by FOLFIRINOX, SABR, and adjuvant chemotherapy. Ongoing adjuvant therapy and monitoring are crucial for younger patients or those with pre-NAT metastasis. Further studies should refine tumor grading systems and explore objective post-NAT evaluation methods.

**Supplementary Information:**

The online version contains supplementary material available at https://doi.org/10.1245/s10434-025-18123-w.

In pancreatic cancer, the involvement of major vessels is a critical factor in determining resectability. Several studies have demonstrated the oncological benefits of neoadjuvant therapy (NAT) for borderline resectable pancreatic cancer.^1–4^ Recently, NAT has been increasingly used for primary resectable pancreatic ductal adenocarcinoma (PDAC) and borderline resectable PDAC, as well as for locally advanced PDAC.^5–7^ However, in evaluating the effect of NAT, imaging methods such as computed tomography (CT) are limited in their ability to differentiate tumors from desmoplasia.^8–11^ Histopathological tumor regression following pancreatic resection with NAT is a definitive indicator of disease response. The presence of tumor cells and the extent of the viable tumor burden are believed to be closely linked to the chemo-response to NAT.^12,13^ Depending on the tumor response to NAT, patient prognosis and the effect of the adjuvant treatment can be predicted. Some studies have reported that a pathological complete response (pCR) is associated with improved prognosis; however, the incidence of pCR varies widely across different centers and NAT regimens.^12–15^ This variability may be owing to the lack of consensus on the methods for pathological assessment following NAT or the choice of NAT regimen. Although various grading systems are used to evaluate the tumor response after NAT, no consensus exists. Currently, the College of American Pathologists (CAP), M.D. Anderson, and Evans grading systems are the most popular tumor response scoring systems.

This study aimed to analyze long-term survival outcomes on the basis of pathologic response using the CAP grade after NAT in PDAC and to identify clinicopathologic factors that influence a favorable pathologic response.

## Patients and Methods

### Study Design and Patients

This retrospective observational study was approved by the Institutional Review Board of Seoul National University Hospital (H-2408-036-1558) and adhered to the Declaration of Helsinki of 1975 and its subsequent revisions. All consecutive adult patients (aged > 18 years) who underwent pancreatic resection after NAT at a single center between January 2009 and December 2023 were included. Patients were initiated on NAT after confirming PDAC diagnosis on the basis of pathological examination. The exclusion criteria included patients without a reported CAP grade (*n *= 32), those who underwent palliative surgery (*n* = 30), and those who received NAT outside the specified protocol (*n* = 8) due to incomplete information.

### Data Collection

The collected demographic information included age, sex, and American Society of Anesthesiologists score. Data on carbohydrate antigen (CA)19-9 were collected after NAT. Similarly, resectability, T stage, lymph node enlargement, and metastasis were reviewed at the time of PDAC diagnosis. Information was collected on the regimens and the duration of NAT. Pathological reports following pancreatic resection were evaluated, and postoperative complications were reviewed.

### Definitions

The CAP grade is a pathological tumor regression grading system. A CAP grade of 0 indicates no viable cancer cells or a complete response. CAP grade 1 means single or rare group of cancer cells. CAP grade 2 refers to residual cancer with evident tumor regression but more than single cells or rare groups of cancer cells. CAP grade 3 indicates extensive residual cancer with no evident tumor regression.^16^ At our center, two dedicated pathologists are solely responsible for the CAP grading assessment. For patients who have undergone NAT, pathological results are systematically reviewed in the multidisciplinary team meeting, where the final assessment is made collectively.

All patients included in this study received neoadjuvant chemotherapy (NAC) or radiotherapy. Neoadjuvant radiotherapy (NAR) included conventional and stereotactic ablative body radiotherapy (SABR). NAC regimens were divided into gemcitabine only, a gemcitabine-based combination (predominantly paclitaxel or capecitabine), and FOLFIRINOX (5-fluorouracil, leucovorin, oxaliplatin, and irinotecan). Modified FOLFIRINOX was included in the FOLFIRINOX group.

The patients underwent pancreatoduodenectomy, distal pancreatectomy, or total pancreatectomy, as determined by tumor location and extent. An R0 resection was defined as a direct margin free of malignant cells. Combined vessel resection was performed if the tumor was presumed to be in a major vessel. Perioperative complications were assessed within 90 days of surgery using the Clavien–Dindo (CD) classification, with CD grades ≥ 3 considered severe complications.

### Follow-up Protocol and Statistical Analyses

The patients underwent regular follow-up assessments at the outpatient clinic, where recurrence was monitored using CA 19-9 levels, and CT scans of the abdomen/pelvis and chest were performed every 3 months. Patient prognosis was evaluated by assessing overall survival (OS) and disease-free survival (DFS). OS was defined as the time from the date of NAT initiation to the date of death or the last follow-up. DFS was defined as the duration between the date of surgery and the occurrence of the first instance of recurrence, death, or the last follow-up date. Data was reviewed starting July 2024.

Continuous variables were expressed as mean values with standard deviation (SD), while categorical variables were presented as total numbers with percentages. Student’s *t*-test and analysis of variance were used to analyze continuous data, whereas categorical variables were assessed using the chi-square and Fisher’s exact tests. The Cox proportional hazards regression model was used to calculate hazard ratios (HR), and 95% confidence intervals (CIs) were used to identify the risk factors associated with shorter OS and DFS. Variables with a *p*-value of < 0.10 in the univariate analysis were included in the multivariate analysis. Statistical significance was set at *p* < 0.05. All statistical analyses were performed using SPSS Statistics for Windows (version 26.0; IBM, Armonk, New York, USA) and R software, version 4.2.3 (R Foundation for Statistical Computing).

## Results

A total of 532 patients underwent pancreatic resection after NAT between January 2009 and December 2023. After applying the exclusion criteria, 462 patients were included in this study. The median follow-up duration after surgery was 16 months (range 0–249). The mean age at the time of operation was 62.8 years (SD ± 9.06), and 50.4% of the patients were male. Clinicopathological features are shown in Table 1, comparing CAP grades 0 and 1 and CAP grades 2 and 3. Regarding the T stage before NAT, the CAP grades 0 and 1 group had a higher proportion of T3 and T4 (52.1%) than the CAP grades 2 and 3 group (33.8%) (*p* < 0.001). The CAP grades 0 and 1 group had a longer duration of NAC (7.38 months versus 6.32 months, *p* = 0.041). After NAT, the preoperative tumor size was smaller in the CAP grades 0 and 1 group, with a smaller number of patients with preoperative CA 19-9 levels >37 U/mL (16.0% versus 42.3%, *p* < 0.001). In addition, the CAP grades 0 and 1 group had a higher R0 resection rate (96.6% versus 86.6%, *p* = 0.001) and lower lymphatic, venous, and perineural invasion.

**Table 1:** Clinicopathological characteristics of patients who received pancreatic resection after neoadjuvant treatment

Variables			All patients (*n* = 462)	CAP 0,1 (*n* = 119)	CAP 2,3 (*n* = 343)	*p*-Value
Patient	Age		62.8 ± 9.06	62.8 ± 8.78	62.8 ± 9.17	0.981
factors	Sex	Male	233 (50.4%)	53 (44.5%)	180 (52.5%)	0.135
	ASA	III-IV	66 (14.3%)	15 (12.6%)	51 (14.9%)	0.551
Pre-NAT	Resectability	Resectable	92 (19.9%)	23 (19.3%)	69 (20.1%)	0.085
factors		Borderline resectable	233 (50.4%)	54 (45.4%)	179 (52.2%)	
		Locally advanced	86 (18.6%)	21 (17.6%)	65 (19.0%)	
		Metastatic	51 (11.0%)	21 (17.6%)	30 (8.7%)	
	Tumor Location	Head	296 (64.1%)	79 (66.4%)	217 (63.3%)	0.580
		Body or Tail	166 (35.9%)	40 (33.6%)	126 (36.7%)	
	Tumor size, mm		31.1 ± 10.72	30.8 ± 12.00	31.2 ± 10.27	0.737
	cT stage	T1, T2	284 (61.5%)	57 (47.9%)	227 (66.2%)	< 0.001
		T3, T4	178 (38.5%)	62 (52.1%)	116 (33.8%)	
	LN enlargement	Yes	104 (22.5%)	32 (26.9%)	72 (21.0%)	0.203
NAT	Neo-adjuvant	FOLFIRINOX	384 (84.2%)	95 (79.8%)	289 (85.8%)	0.196
factors	chemothearpy	Gem + multi agent	55 (12.1%)	20 (16.8%)	35 (10.4%)	
		Gem only	17 (3.7%)	4 (3.4%)	13 (3.9%)	
	NAC duration (m)		6.6 ± 4.90	7.38 ± 5.35	6.32 ± 4.71	0.041
	Neo-adjuvant	No	147 (31.8%)	39 (32.8%)	108 (31.5%)	0.292
	radiotherapy	CCRT	56 (12.1%)	19 (16.0%)	37 (10.8%)	
		SABR	259 (56.1%)	61 (51.3%)	198 (57.7%)	
Post-NAT	Preop tumor size, mm		21.4 ± 8.59	18.3 ± 7.89	22.5 ± 8.58	< 0.001
factors	Preop CA 19-9	> 37UmL	164 (35.5%)	19 (16.0%)	145 (42.3%)	< 0.001
	Vessel resection	Yes	157 (34.0%)	33 (27.7%)	124 (36.2%)	0.094
Postop	Histology	Indeterminate	32 (6.9%)	30 (25.2%)	2 (0.6%)	< 0.001
factors		WD/MD	377 (81.6%)	84 (70.6%)	293 (85.4%)	
		PD/UD	53 (11.5%)	5 (9.4%)	48 (14.0%)	
	Resection status	R0	412 (89.2%)	115 (96.6%)	297 (86.6%)	0.001
		R1	50 (10.8%)	4 (3.4%)	46 (13.4%)	
	Pathologic tumor size, mm		24.2 ± 14.75	11.0 ± 11.27	28.9 ± 12.90	< 0.001
	ypN stage	N0	293 (63.7%)	108 (90.8%)	185 (54.3%)	< 0.001
		N1	140 (30.4%)	10 (8.4%)	130 (38.1%)	
		N2	27 (5.9%)	1 (0.8%)	26 (7.6%)	
	Lymphatic invasion	Yes	95 (20.6%)	5 (4.2%)	90 (26.2%)	< 0.001
	Venous invasion	Yes	129 (27.9%)	7 (5.9%)	122 (35.6%)	< 0.001
	Perineural invasion	Yes	308 (66.7%)	28 (23.5%)	280 (81.6%)	< 0.001
	CD complications	III, IV	77 (16.7%)	25 (21.0%)	52 (15.2%)	0.154
	Adjuvant CTx	Yes	405 (87.7%)	102 (85.7%)	303 (88.3%)	0.517
	Adjuvant RTx	Yes	35 (7.6%)	8 (6.7%)	27 (7.9%)	0.698

CAP, College of American Pathologists; NAT, neoadjuvant treatment; FOLFIRINOX, 5-fluorouracil, irrinotecan, leucovorin, and oxaliplatin; Gem, gemcitabine; NAC, neoadjuvant chemotherapy; NAR, neoadjuvant radiotherapy; CCRT, concomitant chemoradiation therapy; SABR, stereotactic ablative body radiotherapy; CA, carbohydrate antigen; WD, well differentiated; MD, moderately differentiated; PD, poorly differentiated; UD, undifferentiated; CD, Clavien–Dindo; CTx, chemotherapy; RTx, radiotherapy

### Survival Outcomes

**Fig. 1:**
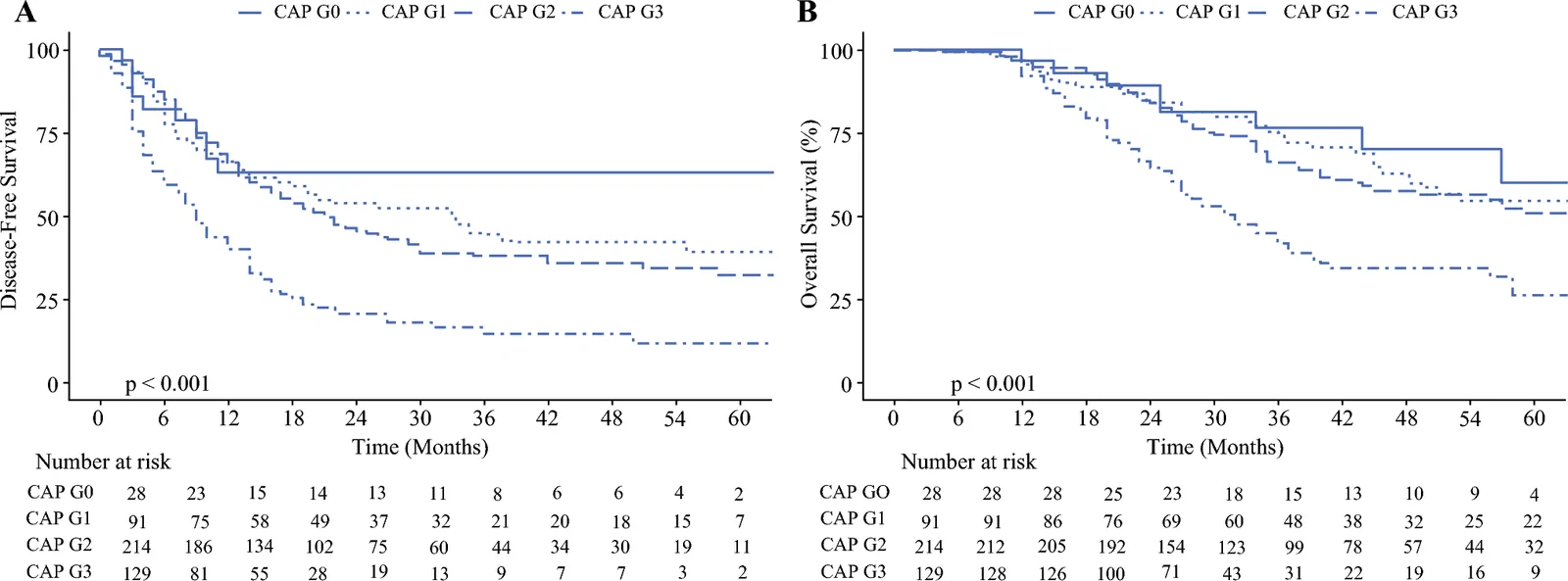
Kaplan–Meier curves of overall survival and disease-free survival depending on the College of American Pathologists (CAP) grades. **A** 5-year disease-free survival (DFS) depending on the CAP grades (5-year survival rate, grade 0 64.3%, grade 1 48.4%, grade 2 42.5%, grade 3 20.2%; *p* < 0.001) **B** 5-year overall survival (OS) depending on the CAP grades (5-year survival rate, grade 0 71.4%, grade 1 69.2%, grade 2 63.6%, grade 3 45.7%; *p* < 0.001)

Only one (0.2%) patient died within 30 days after surgery. Excluding this patient, there were no additional deaths within 90 days postoperatively. The median OS and DFS were 31 months (range 0–249) and 14 months (range 0–100), respectively. The Kaplan–Meier curves for survival outcomes according to the CAP grade are shown in Fig. 1. The DFS (*p* < 0.001) and OS (*p* < 0.001) showed statistically significant differences according to the CAP grade. In patients with a CAP score of 0, the median DFS and OS were not reached. The 5-year DFS rates in patients with CAP grades 0 and 1 were 64.3% and 48.4%, respectively. The 5-year DFS rate in patients with CAP grade 2 was 42.5%, and that of patients with CAP grade 3 was 20.2% (*p* < 0.001, Fig. 1). Compared with CAP grade 3, DFS was superior for CAP grades 0 (64.3% versus 20.2%, *p* < 0.001), 1 (48.4% versus 20.2%, *p* < 0.001), and 2 (42.5% versus 20.2%, *p* < 0.001). Compared with CAP grade 0, CAP grades 1 and 2 had statistically noninferior DFS rates (64.3% versus 48.4%, *p* = 0.273 and 64.3% versus 42.5%, *p* = 0.104, respectively).

For OS, the Kaplan–Meier curves of CAP grades 0, 1, and 2 showed similar tendencies. The 5-year OS rate of CAP grade 3 was 45.7%. Compared with CAP grade 3, CAP grades 0, 1, and 2 had better OS, with 5-year OS rates of 71.4% (*p* = 0.002), 69.2% (*p* < 0.001), and 63.6% (*p* < 0.001), respectively (Fig. 1B).

### Prognostic Factors for DFS and OS

Univariate and multivariate analyses were performed to explore prognostic factors for DFS (Table 2). In the univariate analysis, the risk factors for DFS included NAC regimen, NAC duration, NAR method, preoperative CA 19-9 level, CAP grade, resection status, and adjuvant chemotherapy. Multivariate analysis identified FOLFIRINOX regimen for NAC (HR: 0.54, 95% CI: 0.31–0.94, *p* = 0.031), SABR for NAR (HR: 0.68, 95% CI: 0.52–0.88, *p* = 0.003), preoperative CA 19-9 level > 37 U/mL (HR: 1.45, 95% CI 1.13–1.86, *p* = 0.004), adjuvant chemotherapy (HR: 0.24, 95% CI 0.19–0.31, *p* < 0.001), and R1 resection status (HR: 1.64, 95% CI 1.17–2.31, *p* = 0.005) as the independent factors for DFS. Moreover, CAP grades 2 and 3 (HR: 1.74, 95% CI 1.28–2.67, *p* < 0.001) turned out to be an independent risk factor for shorter DFS.

**Table 2:** Risk factors for disease free survival in patients who received pancreatic resection after neoadjuvant treatment

		Disease free survival					
		Univariate analysis			Multivariate analysis		
	Variables	HR	95% CI	*p*-Value	HR	95% CI	*p*-Value
Patient factors							
Age	≤ 65 (271)	1.00 (Ref)			–	–	–
	> 65 (191)	1.06	0.83–1.34	0.641	–	–	–
Sex	Female (229)	1.00 (ref)			–	–	–
	Male (233)	1.03	0.82–1.30	0.808	–	–	–
ASA	I-II (396)	1.00 (ref)			–	–	–
	III-IV (66)	0.88	0.62–1.24	0.466	–	–	–
Pre-NAT factors							
Resectability	Resectable (92)	1.00 (ref)			–	–	–
	Borderline resectable (233)	1.02	0.75–1.40	0.898	–	–	–
	Locally advanced (86)	0.80	0.55–1.18	0.257	–	–	–
	Metastatis (51)	1.12	0.74–1.71	0.586	–	–	–
NAT factors							
NAC regimen	Gemcitabine only (17)	1.00 (ref)			–	–	–
	Gem + multi agent (51)	0.58	0.32–1.07	0.081	0.73	0.40–1.33	0.299
	FOLFIRINOX (388)	0.42	0.25–0.71	0.001	0.54	0.31–0.94	0.031
NAC duration	≤ 4months (177)	1.00 (ref)			–	–	–
	> 4months (279)	0.73	0.57–0.93	0.009	0.88	0.68-1.13	0.306
NAR	No (147)	1.00 (ref)			–	–	–
	CCRT (56)	1.19	0.83–1.70	0.341	0.67	0.46–1.00	0.048
	SABR (259)	0.75	0.58–0.98	0.031	0.68	0.52–0.88	0.003
Preop factors							
Preoperative	≤ 37U/mL (298)	1.00 (ref)			–	–	–
CA 19-9	> 37U/mL (164)	1.82	1.44–2.30	< 0.001	1.45	1.13–1.86	0.004
Postop factors							
CAP grade	CAP 0, 1 (119)	1.00 (ref)			–	–	–
	CAP 2,3 (343)	1.69	1.26–2.26	< 0.001	1.74	1.28–2.67	< 0.001
R status	R0 (412)	1.00 (ref)			–	–	–
	R1 (50)	1.80	1.30–2.51	< 0.001	1.64	1.17–2.31	0.005
CD	CD I, II (385)	1.00 (ref)			–	–	–
complications	CD III, IV, V (77)	1.13	0.83–1.53	0.432	–	–	–
Adjuvant	N (281)	1.00 (ref)			–	–	–
chemotherapy	Y (181)	0.28	0.22–0.36	< 0.001	0.24	0.19–0.31	< 0.001
Adjuvant	N (427)	1.00 (ref)			–	–	–
radiotherapy	Y (35)	0.92	0.62–1.38	0.689	–	–	–

CAP, College of American Pathologists; NAT, neoadjuvant treatment; FOLFIRINOX 5-fluorouracil, irrinotecan, leucovorin, and oxaliplatin; NAC, neoadjuvant chemotherapy; NAR, neoadjuvant radiotherapy; CCRT, concomitant chemoradiation therapy; SABR, stereotactic ablative body radiotherapy; CA, carbohydrate antigen; WD, well differentiated; MD, moderately differentiated; PD, poorly differentiated; UD, undifferentiated

Regarding OS, male sex, NAC regimen, NAC duration, NAR method, preoperative CA 19-9, CAP grade, resection status, CD complications grade, and adjuvant chemotherapy were included as risk factors in the univariate analysis (Table 3). Multivariate analysis identified the FOLFIRINOX regimen for NAC (HR: 0.40, 95% CI 0.22–0.74, *p* = 0.003), NAC duration > 4 months (HR: 0.56, 95% CI 0.41–0.76, *p* < 0.001), SABR for NAR (HR: 0.67, 95% CI 0.48–0.94, *p* = 0.020), CD complications > III (HR: 1.54, 95% CI 1.05–2.25, *p* = 0.028), and adjuvant chemotherapy (HR: 0.19, 95% CI 0.14–0.26, *p* < 0.001) as the independent factors for OS. Furthermore, CAP grades 2 and 3 (HR: 1.54, 95% CI 1.05–2.25, *p* < 0.028) turned out to be an independent risk factor for shorter OS.

**Table 3:** Risk factors for overall survival in patients who received pancreatic resection after neoadjuvant treatment

		Overall survival						
		Univariate analysis				Multivariate analysis		
	Variables	HR	95% CI	*p*-Value		HR	95% CI	*p*-Value
Patient factors								
Age	≤ 65 (271)	1.00 (Ref)				–	–	–
	> 65 (191)	0.82	0.62–1.10		0.186	–	–	–
Sex	Female (229)	1.00 (ref)				–	–	–
	Male (233)	1.33	1.00–1.76		0.050	0.92	0.68–1.24	0.595
ASA	I-II (396)	1.00 (ref)				–	–	–
	III-IV (66)	0.99	0.66–1.49		0.970	–	–	–
Pre-NAT factors								
Resectability	Resectable (92)	1.00 (ref)				–	–	–
	Borderline resectable (233)	1.07	0.73–1.56		0.743	–	–	–
	Locally advanced (86)	0.76	0.47–1.23		0.257	–	–	–
	Metastatis (51)	1.23	0.75–2.04		0.410	–	–	–
NAT factors								
NAC regimen	Gemcitabine only (17)	1.00 (ref)				–	–	–
	Gem + multi agent (51)	0.63	0.32–1.26		0.191	0.56	0.23–0.92	0.027
	FOLFIRINOX (388)	0.52	0.30–0.90		0.020	0.40	0.22–0.74	0.003
NAC duration	≤ 4months (177)	1.00 (ref)				–	–	–
	> 4months (279)	0.71	0.53–0.95		0.020	0.56	0.41–0.76	< 0.001
NAR	No (147)	1.00 (ref)				–	–	–
	CCRT (56)	1.17	0.78–1.74		0.447	0.80	0.50–1.27	0.337
	SABR (259)	0.73	0.53–1.00		0.053	0.67	0.48-0.94	0.020
Preop factors								
Preoperative	≤ 37U/mL (298)	1.00 (ref)				–	–	–
CA 19-9	> 37U/mL (164)	1.67	1.26–2.23		< 0.001	1.20	0.89–1.63	0.240
Postop factors								
CAP grade	CAP 0, 1 (119)	1.00 (ref)				-	-	-
	CAP 2,3 (343)	1.58	1.12–2.23		0.009	1.84	1.29–2.62	< 0.001
R status	R0 (412)	1.00 (ref)				–	–	–
	R1 (50)	1.66	1.11–2.48		0.013	1.38	0.91–2.11	0.131
Clavien–dindo	CD I, II (385)	1.00 (ref)				–	–	–
complication	CD III, IV, V (77)	1.37	0.95–1.95		0.090	1.54	1.05–2.25	0.028
Adjuvant	N (281)	1.00 (ref)				–	–	–
chemotherapy	Y (181)	0.24	0.18–0.32		< 0.001	0.19	0.14–0.26	< 0.001
Adjuvant	N (427)	1.00 (ref)				–	–	–
radiotherapy	Y (35)	1.29	0.76–2.19		0.345	–	–	–

CAP, College of American Pathologists; NAT, neoadjuvant treatment; FOLFIRINOX 5-fluorouracil, irrinotecan, leucovorin, and oxaliplatin; NAC, neoadjuvant chemotherapy; NAR, neoadjuvant radiotherapy; CCRT, concomitant chemoradiation therapy; SABR, stereotactic ablative body radiotherapy; CA, carbohydrate antigen; WD, well differentiated; MD, moderately differentiated; PD, poorly differentiated; UD, undifferentiated

### Characteristics of Patients with pCR who Experienced Recurrence

In this study, 28 (6.1%) patients achieved pCR, including 9 who experienced recurrence (Supplementary Table 1). Five patients had distant organ recurrence only, four had local recurrence and distant organ recurrence, and none had local recurrence only. The liver was the most common organ with distant metastasis (*n* = 5), followed by the lungs (*n* = 3). Two patients had peritoneal seeding, while one patient had brain metastasis. Local recurrence primarily occurred in the lymph nodes, with only one patient experiencing recurrence in the remaining pancreatic tissue.

Supplementary Table 1 shows the clinicopathological characteristics of patients with pCR who did and did not experience recurrence. The patients with pCR who experienced recurrence were younger than those without recurrence (64.1 versus 56.4, *p* = 0.033). The pCR and recurrence groups had more locally advanced or metastatic pancreatic cancers than those without recurrence (66.7% versus 33.3%, *p* = 0.090). The pCR and recurrence groups had a higher proportion of patients suspected of having distant metastases before NAT (55.6% versus 10.5%, *p* = 0.020). Among those receiving NAC, all patients who achieved pCR without recurrence were treated with FOLFIRINOX (*p* = 0.026). Regarding NAR, a greater percentage of patients with pCR without recurrence received SABR than those with pCR who experienced recurrence (84.2% versus 44.4%, *p* = 0.027).

## Discussion

The use of NAT is increasing in pancreatic cancer regardless of resectability.^1–3,7,17^ Therefore, evaluating the tumor response to NAT is a major concern. However, the lack of preoperative information about cellularity or differentiation makes pathological assessment of the tumor response to NAT very challenging. In imaging studies, tumors are difficult to differentiate from desmoplasias.^18^ Furthermore, a consensus is lacking regarding the NAC regimen, its duration, or criteria for pathological assessment when evaluating the tumor regression grade. However, the increasing use of NAT has changed the treatment of PDAC. There are three widely used tumor regression scoring systems: the four-tier CAP, three-tier MD Anderson, and five-tier Evans grading systems. The CAP grading system is used in many centers.^16,19^ In this study, we aimed to explore the long-term survival outcomes according to the CAP grades and evaluate clinicopathological factors associated with pathologic favorable tumor response.

In DFS, the FOLFIRINOX regimen for NAC, SABR for NAR, preoperative CA 19-9 normalization, R1 resection, and adjuvant chemotherapy turned out to be the independent prognostic factors. The FOLFIRINOX regimen, compared with gemcitabine alone, has been found to have a better effect in many studies.^1,4,17^ It is the standard NAC regimen for patients with PDAC. Likewise, in our study, the FOLFIRINOX regimen showed a better prognosis regarding both DFS (HR: 0.54, 95% CI 0.31–0.94, *p* = 0.031) and OS (HR: 0.40, 95% CI 0.22–0.74, *p* = 0.003). However, compared with gemcitabine alone, gemcitabine-based multiagent regimens showed no statistically significant difference. Regarding NAR, SABR was an independent prognostic factor (HR: 0.68, 95% CI: 0.52–0.88, *p* = 0.003). Many studies have focused on the effects of SABR as NAR.^15^ SABR is a relatively new technique that can deliver higher doses of radiation to the tumor quickly in one to five fractions compared with conventional concomitant radiotherapy. At our center, we routinely use SABR and stereotactic magnetic resonance (MR)-guided on-table adaptive radiation therapy (SMART) for patients with pancreatic cancer. SMART is an MR-guided system that uses the superior soft tissue contrast of MR images acquired before and continuously during treatment delivery. When the tumor is displaced from the correct position, it automatically pauses the treatment. The daily on-table treatment plan can be adapted to account for interfractional anatomical changes.^20^ Using SMART, we can deliver higher doses of radiation about 50 Gy in five fractions with minimized toxicity to adjacent organs. Michael et al. reported that postoperative SMART resulted in favorable long-term OS for patients with locally advanced or borderline resectable pancreatic cancer in a multicenter, open-label phase 2 study.^21^

Although SABR was identified as an independent prognostic factor for both DFS and OS in our multivariate analysis, this finding should be interpreted with caution. SABR is a relatively recent modality that was more frequently utilized in later years of the study period, during which overall treatment protocols, including systemic therapy and perioperative care, have also improved. Therefore, the observed survival benefit may, in part, reflect broader advancements in treatment rather than the effect of SABR alone.

Furthermore, the number of patients receiving SABR, while not negligible, limits the statistical power of subgroup analyses. This raises the possibility that the association between SABR and improved survival may be confounded by patient selection or era-related bias. In light of existing evidence showing mixed results for neoadjuvant radiotherapy, including SABR, further prospective studies with larger sample sizes and controlled designs are necessary to validate these findings.

The FOLFIRINOX regimen for NAC, NAC duration > 4 months, SABR for NAR, CD complications > III, and adjuvant chemotherapy were statistically significant prognostic factors for OS. Particularly, NAC duration > 4 months had a better prognosis for OS (HR: 0.56, 95% CI 0.41–0.76, *p* < 0.001). A duration of over 6 or 8 months did not have a statistically significant effect on survival outcomes. Therefore, in the absence of any evidence of aggravation, NAT for PDAC could be recommended to start with FOLFIRINOX and SABR and last for at least 4 months.^22^ Several randomized controlled trials and meta-analyses have demonstrated that patients with BRPC who undergone NAT, compared with those who undergo upfront surgery, achieve more favorable survival and oncologic outcomes.

NAT is the standard approach for borderline resectable pancreatic cancer. However, there is no consensus on the optimal surgical timing for patients with borderline resectable pancreatic cancer (BRPC) who have undergone NAT. Numerous studies are currently being conducted to determine the ideal duration and regimen for NAT.^3,4^ Our center has also conducted research to identify the optimal duration and regimen. Jung et al. investigated the long-term outcomes of patients with BRPC receiving NAT and proposed an optimal resection timing.^4^ In the FOLFIRINOX group, patients who received more than eight cycles of FOLFIRINOX had a better prognosis (< 6 versus 6–7 versus ≥ 8 cycles). Regarding the optimal duration for radiation therapy, the timing of radiation therapy may vary depending on the scheduling of surgery, and thus, a standardized approach may not be feasible in all cases. Yun et al. reported that patients who received radiotherapy had significantly improved postoperative survival, local control, and R0 resection rates compared with those who did not.^23^ Furthermore, patients who underwent surgery within 4 weeks after completing radiotherapy exhibited lower intraoperative blood loss and a clinically relevant postoperative pancreatic fistula rate compared with those who underwent surgery after more than 4 weeks. As various prospective studies on specific chemotherapy agents are currently underway, additional prospective, multicenter collaborative studies will be needed to determine the optimal treatment duration on the basis of the type of chemotherapy used.

Despite a favorable pathologic tumor regression response, adjuvant chemotherapy remains an independent prognostic factor for both DFS and OS (DFS, HR: 0.24, 95% CI 0.19–0.31, *p* < 0.001; OS, HR: 0.19, 95% CI 0.14–0.26, *p* < 0.001). NAC also increased outcomes, but in cases of recurrence within the pCR group, distant organ metastases were observed. Although the patients had a good pathological tumor regression response, adjuvant chemotherapy improved their prognosis, likely owing to its ability to target undetected residual disease. Tumor regression response to NAT could also provide some clues about the kind of adjuvant treatment that should be administered and how long it should be continued^14^.

OS and DFS were significantly associated with the CAP grade (OS, *p* < 0.001; DFS, *p* < 0.001; Fig. 1). CAP grade 0 did not reach the median survival. Patients with CAP grade 1 had a median OS of 72 months and a median DFS of 33 months. Similarly, patients with CAP grade 2 had a median OS of 64 months and a median DFS of 21 months. Moreover, those with CAP grade 3 had a median OS of 32 months and a median DFS of 9 months. Patients with CAP grades 0, 1, and 2 had significantly better survival outcomes than those with CAP grade 3 (CAP grade 0, *p* = 0.002; CAP grade 1, *p* < 0.001; and CAP grade 2, *p* < 0.001). However, CAP grade 0 showed no significant difference in DFS compared with CAP grades 1 or 2 (CAP grade 1, *p* = 0.273; CAP grade 2, *p* = 0.104), which can be primarily attributed to the small number of patients in the CAP grade 0 group (n=28). Prior to NAT, patients with CAP grade 0 had a higher proportion in locally advanced and metastatic pancreatic cancer compared with those with CAP grade 1 and 2 (CAP grade 0 42.9%; CAP grade 1 34%; CAP grade 2 29.5%). For this reason, among the patients who achieved pCR but later experienced recurrence, there was a tendency for recurrence to occur as distant metastasis rather than from the primary lesion. This could be another contributing factor to the lack of a significant difference in DFS between CAP grade 0 and grade 1/2.

Patients with pCR who experienced recurrence tended to be younger and more likely to have had metastases before NAT. In contrast, the pCR group without recurrence received the FOLFIRINOX regimen for NAC and underwent SABR more frequently than the group with recurrence. FOLFIRINOX and SABR as NAT decreased the tumor recurrence rate. Patients with pCR who experienced recurrence had a higher proportion of locally advanced or metastatic pancreatic cancer before NAT.

When analyzing the recurrence patterns in patients with pCR, no cases of isolated local recurrence were observed, and most recurrences involved distant metastases. Patients who achieved pCR had a higher proportion of pre-NAT metastases and a greater prevalence of T3 and T4 tumors. These findings suggest that while pCR may indicate effective control of the primary tumor and local disease, it may not fully account for the presence of distant metastases or dormant tumor cells.

In this study, CAP grade was identified as an independent prognostic factor for survival outcomes. However, the CAP grading system has some limitations. First, it is controversial in terms of reliability. Incidence of CAP grade 0 and pCR is reported to vary widely across centers from 3% to 11%.^13^ Evaluating tumor regression response by the postoperative specimen is challenging as specimen handling differs between centers, and PDAC often contains skipped lesions. Boris et al. reported that the CAP grade had moderate reliability, with inconsistencies in pathologic reports among pathologists examining the same specimen.^6^ Although this was a single-center study, which may reduce the challenges associated with specimen handling, the limitation of the CAP grading system remains. Many studies have attempted to achieve this, but no consensus exists on the grading system.^6,12,19,24–26^ For example, Lee et al. proposed three-tier histologic tumor regression grading scheme modified from CAP grade.

The CAP grading system has a limitation that can be achieved only after the surgery. It is very important to evaluate the tumor response after NAT because it can be the guideline for timing to do surgery or change the chemotherapy regimen. Usually, to evaluate the tumor response for NAT, Response Evaluation Criteria in Solid Tumours (RECIST) is used. But in pancreatic cancer, tumors are difficult to differentiate from desmoplasias after NAT.^18^ So many studies have been done for finding the tools to evaluate the effect of NAT before the surgery. For example, some trials have explored imaging tools such as 2-fluoro-2-deoxy-D-glucose positron emission tomography/CT or diffusion-weighted magnetic resonance (MR) imaging to evaluate the response in PDAC after NAT.^9–11^ In locally advanced rectal cancer, the addition of diffusion weighted MR imaging to conventional T2 weighted sequences turned out to improve the diagnostic performance in complete response.^27^ In addition, many studies have been conducted on CA 19-9 as biomarkers for the remnant tumor.^28^

The third limitation is that most current grading systems, including the CAP grade, are predominantly qualitative. The qualitative evaluation system can be used to assess the effect of NAT or predict patient prognosis but has limitations in detecting residual disease. Therefore, it is necessary to develop quantitative evaluation systems to assess tumor regression responses systematically. Some studies have attempted to develop a tumor regression grading system for other cancers.^27,29^ Johanna et al. reported an artificial intelligence-based tool for quantitative deciphering of colorectal cancer.^30^ Sara et al. found that the molecular characterization of pretreatment biopsies using RAS, RAF, and SMAD4 in patients with locally advanced rectal cancer after NAT had the potential to identify high-risk patients.^29^ Hiroshi et al. examined the association between breast cancer resistance protein expression and prognosis in primary breast tumors after NAC using quantitative immunohistochemical analysis.^31^ Shintia et al. studied the association between clinical response with percentage of apoptosis to evaluate pathologic responses both quantitatively and qualitatively in breast cancers.^32^ In pancreatic cancer, Matsuda et al. suggested new grading systems, including a five-tiered system based on the area of residual tumor.^19^ Further studies will be needed to develop an objective tool for quantitative evaluation of tumor regression in pancreatic cancer.^19^

In conclusion, patients with favorable CAP grades after NAT for PDAC generally exhibit better survival outcomes. Factors associated with CAP grades 0 and 1 after NAT include the FOLFIRINOX regimen and SABR. If the patient is younger or has metastasis before NAT, adjuvant treatment should be followed, and close monitoring is essential. The current tumor grading systems remain controversial and lack consensus. Further studies are required to develop a new, objective grading system for pancreatic cancer after NAT.

## Supplementary Information

Below is the link to the electronic supplementary material.

Supplementary file1 (DOCX 15 kb)
