# Supplementary material for: Evaluating Tumor Regression and Survival Outcomes in Pancreatic Ductal Adenocarcinoma After Neoadjuvant Treatment according to CAP Grading System: Clinical Usefulness and Limitations
Source: Ann Surg Oncol. 2025 Aug 26;32(13):9956–65. doi: 10.1245/s10434-025-18123-w (PMC12589204; doi:10.1245/s10434-025-18123-w)
Supplement: Supplementary file 1 — Supplementary file1 (DOCX 15 kb) [file 10434_2025_18123_MOESM1_ESM.docx]

Supplementary Table 1. Clinico-pathological characteristics of patients who had CAP grade 0 after pancreatic resection.

| Variables | | | pCR without recurrence (n=19) | pCR with recurrence (n=9) | p-value |
| --- | --- | --- | --- | --- | --- |
| Patient | Age |  | 64.1 ± 8.71 | 56.4 ± 7.52 | 0.033 |
| factors | Sex | Female | 10 (52.6%) | 4 (44.4%) | 1.000 |
|  |  | Male | 9 (47.4%) | 5 (55.6%) |  |
|  | ASA | I-II | 15 (78.9%) | 8 (88.9%) | 1.000 |
|  |  | III-IV | 4 (21.1%) | 1 (11.1%) |  |
| Pre-NAT | Resectability | Resectable / Borderline resectable | 13 (68.4%) | 3 (33.3%) | 0.090 |
|  |  | Locally advanced / Metastatic | 6 (31.6%) | 6 (66.7%) |  |
|  | Tumor size, mm |  | 32.6 ± 9.77 | 29.3 ± 7.23 | 0.389 |
|  | Pre NAT T stage | T1, T2 | 10 (52.6%) | 5 (55.6%) | 1.000 |
|  |  | T3, T4 | 9 (47.4%) | 4 (44.4%) |  |
|  | LN enlargement | No | 13 (68.4%) | 9 (100.0%) | 0.136 |
|  |  | Yes | 6 (31.6%) | 0 (0.0%) |  |
|  | Pre NAT metastasis | No | 17 (89.5%) | 4 (44.4%) | 0.020 |
|  |  | Yes | 2 (10.5%) | 5 (55.6%) |  |
|  | Pre NAT CA 19-9 | ≤ 37U/mL | 10 (52.6%) | 2 (22.2%) | 0.223 |
|  |  | > 37UmL | 9 (47.4%) | 7 (77.8%) |  |
| NAT | Neo-adjuvant | FOLFIRINOX | 19 (100.0%) | 6 (66.7%) | 0.026 |
| factors | chemothearpy | Gemcitabine + multi agent | 0 (0.0%) | 2 (22.2%) |  |
|  | regimen | Gemcitabine only | 0 (0.0%) | 1 (11.1%) |  |
|  | Neo-adjuvant | No | 1 (5.3%) | 4 (44.4%) | 0.027 |
|  | radiotherapy | CCRT | 2 (10.5%) | 1 (11.1%) |  |
|  |  | SABR | 16 (84.2%) | 4 (44.4%) |  |
| Post-NAT | Pre-operative | ≤ 37U/mL | 18 (94.7%) | 8 (88.9%) | 1.000 |
| factors | CA 19-9 | > 37UmL | 1 (5.3%) | 1 (11.1%) |  |
| CR, complete response; NAT, neoadjuvant treatment; FOLFIRINOX, 5-FU, irrinotecan, leucovorin, and oxaliplatin; NAC, neoadjuvant chemotherapy; NAR, neoadjuvant radiotherapy; CCRT, concomitant chemoradiation therapy ; SABR, stereotactic ablative body radiotherapy; CA, carbohydrate antigen; | | | | | |
